# Neuroprotective effects of a medium chain fatty acid, decanoic acid, isolated from *H. leucospilota* against Parkinsonism in *C. elegans* PD model

**DOI:** 10.3389/fphar.2022.1004568

**Published:** 2022-12-13

**Authors:** Tanatcha Sanguanphun, Nilubon Sornkaew, Nawaphat Malaiwong, Pawanrat Chalorak, Prapaporn Jattujan, Nakorn Niamnont, Prasert Sobhon, Krai Meemon

**Affiliations:** ^1^ Department of Anatomy, Faculty of Science, Mahidol University, Bangkok, Thailand; ^2^ Department of Chemistry, Faculty of Science, King Mongkut’s University of Technology Thonburi, Bangkok, Thailand; ^3^ Department of Radiological Technology and Medical Physics, Chulalongkorn University, Bangkok, Thailand; ^4^ Department of Anatomy, Faculty of Medicine, Khon Kaen University, Khon Kaen, Thailand; ^5^ Center for Neuroscience, Faculty of Science, Mahidol University, Bangkok, Thailand

**Keywords:** Parkinson’s disease, dopaminergic neurons, α-synuclein, *Holothuria leucospilota*, *C. elegans*, decanoic acid

## Abstract

Sea cucumbers are marine organism that have long been used for food and traditional medicine in Asian countries. Recently, we have shown that ethyl acetate fraction (HLEA) of the crude extract of the black sea cucumber, *Holothuria leucospilota,* could alleviate Parkinsonism in *Caenorhabditis elegans* PD models. In this study, we found that the effective neuroprotective activity is attributed to HLEA-P1 compound chemically isolated and identified in *H. leucospilota* ethyl acetate. We reported here that HLEA-P1 could attenuate DAergic neurodegeneration, improve DAergic-dependent behaviors, reduce oxidative stress in 6-OHDA-induced *C. elegans*. In addition, HLEA-P1 reduced α-synuclein aggregation, improved behavior deficit and recovered lipid deposition in transgenic *C. elegans* overexpressing α-synuclein. We also found that HLEA-P1 activates nuclear localization of DAF-16 transcription factor of insulin/IGF-1 signaling (IIS) pathway. Treatment with 25 μg/ml of HLEA-P1 upregulated transcriptional activity of DAF-16 target genes including anti-oxidant genes (such as *sod-3*) and small heat shock proteins (such as *hsp16.1*, *hsp16.2*, and *hsp12.6*) in 6-OHDA-induced worms. In α-synuclein-overexpressed *C. elegans* strain, treatment with 5 μg/ml of HLEA-P1 significantly activated mRNA expression of *sod-3* and *hsp16.2.* Chemical analysis demonstrated that HLEA-P1 compound is decanoic acid/capric acid. Taken together, our findings revealed that decanoic acid isolated from *H. leucospilota* exerts anti-Parkinson effect in *C. elegans* PD models by partly modulating IIS/DAF-16 pathway.

## 1 Introduction

Parkinson’s disease (PD) is a chronic neurodegenerative disease characterized by the selective degeneration of the dopaminergic (DAergic) neurons in substantia nigra pars compacta (SNc) and the aggregation of the misfolded α-synuclein protein within Lewy body. The degeneration of DAergic neurons contributes to the depletion of the neurotransmitter, dopamine, in the striatal neurons, resulting in motor impairment (Maiti et al., 2017). The etiologic causes of PD are genetic mutations, environmental toxins, and aging (Maiti et al., 2017). Although the precise molecular pathogenesis behinds PD remains to be elucidated, oxidative stress has been proposed as the major pathogenic event causing PD (Moore et al., 2005). Based on the Global Burden of Disease study, PD is considered as a neurological disorder with the fastest growing prevalence (Feigin et al., 2017). Unfortunately, this disease is still incurable. Currently, the conventional treatments are based on the symptomatic relief (Maiti et al., 2017). In this regard, exploring therapeutic approaches that can slow down or stop the degenerative process due to PD has become an urgent issue.

Recently, bioactive compounds derived from marine organisms have gained attention in the field of drug discovery for neurodegenerative diseases including PD (Huang et al., 2019). Sea cucumbers are among marine animals with high economic, food, and medicinal values, especially in Asian countries (Mashjoor et al., 2018). From a nutritional perspective, the sea cucumbers contain several bioactive ingredients such as peptides, triterpene glycosides, fucoidan, and essential fatty acids (Xu et al., 2018). In this regard, a number of biological and therapeutics properties including anti-oxidation, anti-inflammation, anti-cancer, as well as anti-neurodegeneration have been ascribed to substances obtained from various species of sea cucumbers (Xu et al., 2018). In particular to PD, crude extracts and bioactive compounds from sea cucumbers have been shown to protect neurotoxin-induced DAergic neurodegenerations in several studies. For example, ethyl acetate and butanol fractions of the crude extract of *Holothuria scabra* (*H. scabra*) and their purified compounds, diterpene glycosides, could significantly reduce DAergic neurodegeneration and inhibit abnormal aggregation of α-synuclein in *Caenorhabditis elegans* (*C. elegans*) PD models (Chalorak et al., 2018; Chalorak et al., 2021). In addition to *H. scabra*, the black sea cucumber, *H. leucospilota*, is found to be predominant throughout the Indo-Pacific region including Thailand, and it is one of commercial species in Southeast Asia (Choo et al., 2008). Similar to other sea cucumber species, *H. leucospilota* also contains several bioactive compounds such as phenols, terpenoids, flavonoid, saponin, glycoside, steroids, essential fatty acids (Ceesay et al., 2019). To ascertain nutritional property and enhance economic value of *H. leucospilota*, the neuroprotective effects of the extracts from this species were investigated. Recent study from our group demonstrated that ethyl acetate fraction of the crude extract of *H. leucospilota* (HLEA) mediates anti-Parkinson’s activity by attenuating DAergic neurodegeneration and inhibiting α-synuclein aggregation in the *C. elegans* PD models (Malaiwong et al., 2019). However, the specific compounds with anti-PD property in such fraction has not yet been elucidated. Thus, in the present study we identified and isolated the active compounds from HLEA, and evaluate their anti-PD effects as well as investigate possible mechanisms of action of these compounds. Hopefully, the obtained data would substantiate the nutraceutical value of *H. leucospilota* bioactive compounds and establish them as potential candidate pharmaceuticals for anti-PD.

To investigate the anti-PD activity of *H. leucospilota* compounds, *C. elegans* was used as a model organism. *C. elegans* provides several advantages for being used as an animal model including its short life cycle and high reproductive rate. Besides, *C. elegans* has 8 DAergic neurons in its nervous system (Harrington et al., 2010), that could be selectively induced to undergo neurodegeneration by the neurotoxin such as 6-hydroxydopamine (6-OHDA) (Nass et al., 2002). Moreover, the transgenic *C. elegans* NL5901 expressing human α-synuclein has been created for studying the effects and mechanisms of α-synucleinopathies (Bodhicharla et al., 2012). Therefore, our study aimed to investigate the neuroprotective effects and underlying mechanism of the isolated compounds from HLEA against PD using *C. elegans* as a model.

## 2 Materials and methods

### 2.1 Collection and extraction of *H. leucospilota*

The black sea cucumber *H. leucospilota* were collected from Prachuap Khiri Khan province, and its species were identified by experts from Prachuap Khiri Khan Coastal Fisheries Research and Development Center, Thailand. The collected samples were then cleaned by distilled water and anesthetized by cooling in ice. The body walls of *H. leucospilota* were dissected, cut into small pieces, and stored at −80°C. Then, the samples were freeze-dried using FreezeDry Supermodulyo-230. The freeze-dried *H. leucospilota* samples (1.2 kg) were ground to powder and macerated with *n*-hexane. After evaporation of the solvent and reduction of pressure, *n*-hexane fraction (HLHE, 3.2 g) and residue were obtained. The acquired residue was then extracted by ethyl acetate (EtOAc) to obtain HLEA fraction (3.5 g). The HLEA fraction was subsequently subjected to chemical isolation and identification. The procedures for handling the sea cucumbers were ethically approved by Mahidol University-Institute Animal Care and Use Committee (MU-IACUC; MUSC60-049-399).

### 2.2 Isolation and structural characterization of chemicals in *H. leucospilota* ethyl acetate (HLEA) fraction

In the isolation process, the HLEA fraction was purified sequentially by solvent partition, silica-gel column chromatography (CC) and Thin Layer Chromatography (TLC). In this study, CC was carried out using Merck silica gel 60 (finer than 0.063 mm) and Pharmacia Sephadex LH-20. For TLC, Merck precoated silica gel 60 F_254_ plates were used. Spots on TLC were detected under the UV light and by spraying with anisaldehyde-H_2_SO_4_ reagent followed by heating. The HLEA fraction (2.0 g) was fractionated by CC using Sephadex LH-20 condition MeOH 100%, and the eluates were examined by the TLC to finally obtain 3 subfractions (EA1-EA3). Then, 101 mg of fraction EA1 was subjected to CC using n-hexane-EtOAc (80:20) to afford compound 1 (HLEA-P1, 25.8 mg). 135 mg of Fraction EA2 was subjected to CC using n-hexane-EtOAc (80:20) to afford compound 2 (HLEA-P2, 20.2 mg) and compound 3 (HLEA-P3, 18.3 mg). 258 mg of Fraction EA3 was isolated by CC using n-hexane-EtOAc (70:30) to yield compound 4 (HLEA-P4, 20.5 mg), compound 5 (HLEA-P5, 15.1 mg) and compound 6 (HLEA-P6, 20.1 mg). Chemical structures of the derived compounds were analysed by ^13^C/^1^H- NMR. In this study, ^1^H and ^13^C NMR were recorded on a Bruker AVANCE 400 FT-NMR spectrometer operating at 400 (^1^H) and 100 (^13^C) MHz. The high resolution mass spectra were obtained using Bruker micrOTOF-QII mass spectrometer.

### 2.3 *C. elegans* strains and maintenance

The *C. elegans* strain used in this study were: N2, Bristol (wild-type), BY250 strain (*vtIs7; dat-1p::GFP*), NL5901 strain [*pkIs2386, unc-54p::α-synuclein::YFP + unc-119(+)*], CF1553 strain [*muIs84*, (*pAD76*) *sod-3p::GFP + rol-6(su1006)*], CL2166 strain [*dvIs19*, (*pAF15*)*gst-4p::GFP::NLS*] and TJ356 strain [*daf-16p::daf-16a/b::GFP + rol-6(su1006)*]. All strains were provided by the *Caenorhabditis* Genetics Center (CGC), University of Minnesota, Minneapolis, United States), except BY250 which was obtained from Prof. Dr. Randy Blakely, Florida Atlantic University, United States. All strains were cultured in nematode growth medium (NGM) plate containing *Escherichia coli* (*E. coli*) strain OP50 as a food source and maintained in 20°C incubator. All *C. elegans* experiments were ethically performed under the guidelines of Faculty of Science, Mahidol University–Institutional Animal Care and Use Committee (MUSC–IACUC; MUSC60-048-398).

### 2.4 Preparation of *H. leucospilota* compounds

HLEA compounds were dissolved in dimethyl sulfoxide (DMSO). Stock solution of HLEA compounds at different doses were prepared and stored at −20°C. To deliver the HLEA compounds to *C. elegans*, HLEA substances were mixed with *E. coli* OP50 to obtain the final concentrations at 1, 5, 25, and 50 μg/ml in 1%DMSO (v/v).

### 2.5 Worm synchronization

To obtain the synchronous population of worms, the gravid adult worms were exposed with the bleaching solution (12% NaClO and 10% 1 M NaOH) with subsequent shaking on a mini-tube rotator for 10–12 min. Then, eggs were separated by centrifugation at 4,000 rpm for 90 s. To stop bleaching reaction, the supernatant was discarded, followed by washing the pellet three times by M9 buffer. Eggs were added with 1 ml of M9 buffer, transferred to NGM plate without OP50 and incubated overnight at 20°C to obtain newly hatched L1 larvae. For experiments, the synchronized L1 larvae were placed on the new NGM plates containing OP50 and incubated at 20°C and allowed to grow to L3 larvae.

### 2.6 6-OHDA-induced DAergic neurodegeneration assay

In this study, a neurotoxin 6-OHDA was used for selectively destroying DAergic neurons of *C. elegans,* according to a previous method (Nass et al., 2002). Synchronized L3 larvae were incubated in a solution containing 50 mM 6-OHDA, 10 mM ascorbic acid and diluted OP50. The worms were exposed to the assay solution for 1 h at 22°C and gently stirred every 10 min. After 1 h of incubation, worms were washed three times by M9 buffer. The 6-OHDA-induced worms were transferred to NGM plates containing *E. coli* OP50 with 1, 5, 25, and 50 μg/ml of HLEA compounds for 72 h at 20°C. For untreated group, 6-OHDA-treated worms were incubated in NGM plates containing OP50 mixed with 1% DMSO (v/v). After 72 h of treatment, the worms were treated in various assays and observed as described below.

### 2.7 Analysis of the viability of DAergic neurons

To evaluate the viability of DAergic neurons, BY250 (*vtIs7; dat-1p::GFP*) were used in this assay. After 72 h of treatment, the viability of DAergic neurons of BY250 was evaluated by measuring GFP fluorescence intensity of DAergic cephalic neurons (CEPs). Worms were washed three times by M9 buffer, transferred to 2% agarose pad on a glass slide. Then, worms were anesthetized with 30 mM sodium azide and finally covered with a coverslip. Imaging of head region of BY250 was taken using a fluorescence microscope (BX53; Olympus Corp., Tokyo, Japan) and fluorescence intensity of CEP neurons was quantified using ImageJ software (National Institute of Health, NIH, Bethesda, MD, United States).

### 2.8 Basal slowing assay

The assay was performed to assess the function of DAergic neurons in *C. elegans*. In the presence of food, the well-fed worms move slower than the starved worms. This dopamine-dependent behavior is known as the basal slowing response (Sawin et al., 2000). After exposure to 6-OHDA and incubation with the extracts for 72 h, N2 wild-type worms were washed by M9 buffer to remove the remaining food in their bodies. Worms were then transferred to the NGM plates with or without OP50 bacteria. To avoid overstimulation of the sensing response, worms were settled for 5 min before recording with video camera for 20 s. Then, their body bendings were counted and the basal slowing rate was calculated as previously described (Malaiwong et al., 2019). The basal slowing rate = 100—locomotory rate (%), when the locomotory rate (%) = (rate of bending in the presence of bacteria/rate of bending in the absence of bacteria) ×100.

### 2.9 Ethanol avoidance assay

In *C. elegans*, DAergic neurons are responsible for ethanol avoidance behavior (Lee et al., 2009). The assay plates were prepared by dividing a 9 cm NGM plate into four quadrants with an inner circle with radius 0.5 cm around the center of the plate and then marked the top left (A) and bottom right quadrants (D) as control quadrants and the top right (B) and bottom left (C) quadrants as ethanol quadrants. In this assay, NGM with 2.5% agar was used to prevent the worms from digging inside the agar. To start the ethanol avoidance assay, 50 μl ethanol was added into two quadrants (B and C) of assay plate. The 6-OHDA-treated worms which were cultured with or without the compounds for 72 h were washed by M9 buffer. Next, 20 μl of the suspension with 50–100 worms was placed on the center of the assay plate with rapid removal of the buffer from the plate. The worms were then allowed to move for 30 min at 25°C. The number of worms in each quadrant were counted. The worms that do not cross the inner circle were not scored. Ethanol avoidance was calculated using the formula: Ethanol avoidance index = [(number of worms in control quadrants)—(number of worms in ethanol quadrants)]/total number of worms (Cooper et al., 2015).

### 2.10 Analysis of α-synuclein aggregation

To evaluate the level of α-synuclein aggregation, transgenic worms strain NL5901 [*pkIs2386, unc-54p::α-synuclein::YFP + unc-119(+)*] were used. In NL5901 strain, YFP tagged-human α-synuclein is expressed under the body wall muscle-specific promoter, allowing visualization of α-synuclein in the body wall muscle. Briefly, synchronized NL5901 L3 worms were transferred to NGM plates containing *E. coli* OP50 with different doses of HLEA-P1 for 72 h at 20°C. For untreated group, worms were incubated in NGM/OP50 containing only 1% (v/v) DMSO. After 72 h of treatment, worms were washed three times with M9 buffer and transferred to the 2% agarose pad on a glass slide for fluorescence imaging. Images of the whole bodies of NL5901 was taken by using a fluorescence microscope (BX53; Olympus Corp., Tokyo, Japan) and the YFP intensity of the aggregated α-synuclein was quantified using ImageJ software (National Institute of Health, NIH, Bethesda, MD, United States).

### 2.11 Thrashing behavior assay

Aggregation of α-synuclein has been reported to associate with impaired thrashing behavior in *C. elegans*. Thrashing behavior can be assessed by counting the number of body bending in liquid media as described previously (Pujols et al., 2018). In this assay, wild-type N2 and NL5901 [*pkIs2386, unc-54p::α-synuclein::YFP + unc-119(+)*] were used. After incubation with HLEA-P1, adult worms at day 3 or day 5 were placed on clean NGM plates with M9 buffer. The worms were allowed to settle for 1 min to avoid over stimulation by stress. Then, the number of body bendings by worms were tracked by video-recording for 30 s in each condition. Thrashing rate was analyzed using wrMTrck plugin for ImageJ (National Institute of Health, NIH, Bethesda, MD, United States) and represented as body bendings per second (bbps).

### 2.12 Nile red staining

Intracellular lipid in *C. elegans* was measured by Nile red staining as described previously (Malaiwong et al., 2019). Wild-type N2 and transgenic NL5901 worms were used in this assay. A stock solution of Nile red at concentration 0.5 mg/ml of acetone was prepared and further diluted in *E. coli* OP50 at a ratio of 1:250. Synchronized NL5901 L3 larvae were incubated on NGM containing *E. coli* OP50/Nile red with different doses of HLEA-P1 for 72 h at 20°C. After incubation, worms were washed three times by M9 buffer, transferred to 2% agarose pad on a glass slide. Then, worms were anesthetized with 30 mM sodium azide and finally enclosed with a coverslip. Lipid deposition in worms was observed under a fluorescence microscope (BX53; Olympus Corp., Tokyo, Japan). The lipid profiles were assessed by determining the average intensity of Nile red fluorescence from the whole bodies of worms using ImageJ software (National Institute of Health, NIH, Bethesda, MD, United States).

### 2.13 Measurement of intracellular ROS levels

Intracellular ROS was measured using 2′,7′-Dichlorodihydrofluorescein diacetate (H_2_DCF-DA) probe by following the protocol described in a previous study (Yoon et al., 2018) with minor modifications. Briefly, after exposure to 6-OHDA and incubation with the compounds for 72 h, the worms were harvested and washed three times in M9 buffer. Then, 30 worms were transferred into a black 96-well plate containing 50 μl M9 buffer by using the platinum wire (5 worms/well, 6 wells for each treatment). A 50 μl of H_2_DCF-DA (final concentration, 25 μM in M9) was added to each well and the samples were incubated at room temperature for 1 h. Then, the plate was inserted into microplate Fluorescence reader with the excitation at 485 nm and emission at 530 nm. Samples were read after 1 h of incubation. The ROS level was calculated as the DCF fluorescence signal (Li et al., 2016). Each condition was performed independently three times.

### 2.14 Analysis for SOD-3 and GST-4 expression

In this assay, *C. elegans* strain CF1553 [*muIs84, (pAD76) sod-3p::GFP + rol-6(su1006)*] and CL2166 [*dvIs19,* (*pAF15*)*gst-4p::GFP::NLS*] were used. CF1553 and CL2166 are transgenic *C. elegans* strains expressing SOD-3 and GST-4 as a reporter gene, respectively. GFP-tagged SOD-3 can be observed in the head, tail and vulva of the CF1553. For CL2166, GFP-tagged GST-4 protein can be visualized in the head region, body wall muscle, intestine. Briefly, synchronized L3 larvae were exposed to 6-OHDA and then fed with different doses of HLEA-P1 as described in 6-OHDA-induced DAergic neurodegeneration assay. After 72 h of treatment, worms were washed three times with M9 buffer and transferred to 2% agarose pad on a glass slide for fluorescence imaging. The GFP intensity of SOD-3:GFP and GST-4:GFP was quantified using ImageJ software (National Institute of Health, NIH, Bethesda, MD, United States).

### 2.15 Analysis for DAF-16 nuclear localization

To observe subcellular localization of DAF-16 transcription factor, TJ356 [*daf-16p::daf-16a/b::GFP + rol-6(su1006)*] in which GFP-tagged DAF-16 protein were used in this assay. Synchronized L3 larvae were exposed to 6-OHDA and supplemented with different doses of HLEA-P1 as described previously. After 72 h of treatment, worms were washed three times with M9 buffer and transferred to 2% agarose pad on a glass slide for fluorescence imaging under a fluorescence microscope (BX53; Olympus Corp., Tokyo, Japan). Subcellular localization of DAF-16 was categorized as nuclear, intermediate and cytosolic. In this study, worms exhibited strong nuclear fluorescence dots were counted as nuclear. Worms having no fluorescence dot in cytoplasm were counted as cytosolic, while worms exhibited unclear cytosolic DAF-16 and unclear nuclear fluorescence dots were assigned as intermediate. All conditions were performed in three independent replicates. In each replicate, approximately 30 worms were used.

### 2.16 Reverse transcription-quantitative polymerase chain reaction (RT-qPCR)

Approximately 800–1,000 of 6-OHDA-induced worms and NL5901 worms overexpressing α-synuclein were treated with 5 and 25 μg/ml HLEA-P1 for 72 h. In untreated group, worms were incubated in NGM/OP50 containing 1% (v/v) DMSO without HLEA-P1. After 72 h, all worms were washed by 3A water three times, centrifuged and collected. Total RNA was extracted using RNA extraction kit (Qiagen, Germany) following the manufacture’s protocol and kept at −80°C. Then, total RNA was converted into complementary DNA (cDNA) with iScript™ Reverse transcriptase Supermix for RT-qPCR (Bio-Rad, CA, United States). RT-qPCR was performed using SsoFast™ EvaGreen^®^ Supermix with Low ROX qRT-PCR (Bio-Rad, CA, United States). Forward and reverse primers for specific genes were shown in Table 1. Real-time PCR were performed by holding the samples at 95°C for 30 s. Then, PCR samples were set for denaturing at 95°C for 5 s and to annealing at 60°C for 30 s. After 44 cycles, the sample were then heated up to 95°C. Eventually, EvaGreen fluorescence were detected by Real time PCR detection system (Bio-Rad, United States) and Cq values were obtained. The Cq values of control and treated groups were then calculated *via* 2^−(ΔΔCq)^ equation representing fold change in the expression of each gene. The relative mRNA expression levels were normalized using reference internal control gene, *act-1.*

**TABLE 1:** Primer sequences used for qRT-PCR analysis.

*C. elegans* genes	NCBI accession number	Primer	Primer sequences (5′ to 3′)
*sod-3*	NM_078363.9	F	AGC​ATC​ATG​CCA​CCT​ACG​TGA
		R	CAC​CAC​CAT​TGA​ATT​TCA​GCG
*hsp-16.1*	NM_072956.4	F	CGT​CCA​GCT​CAA​CGT​TCT​GT
		R	TGG​CTT​GAA​CTG​CGA​GAC​AT
*hsp-16.2*	NM_001392482.1	F	GTC​ACT​TTA​CCA​CTA​TTT​CCG​T
		R	CAA​TCT​CAG​AAG​ACT​CAG​ATG​G
*hsp-12.6*	NM_069267.4	F	TGG​TTC​CTT​CGT​CAG​CCA​TC
		R	GGC​ATT​TCC​TCC​AAG​ACT​AAC
*act-1*	NM_073418.9	F	ATC​GTC​ACC​ACC​AGC​TTT​CT
		R	CAC​ACC​CGC​AAA​TGA​GTG​AA

### 2.17 Statistical analysis

All experiments were performed in three independent trials. The data were statistically analyzed by GraphPad Prism Software. Significant difference between treatment groups and untreated group (1%DMSO) were compared using two-way ANOVA. *p*-value < .05 was considered significant.

## 3 Results

### 3.1 Effect of HLEA compounds 1–6 against 6-OHDA-induced DAergic neurodegeneration in *C. elegans* PD model

**FIGURE 1:**
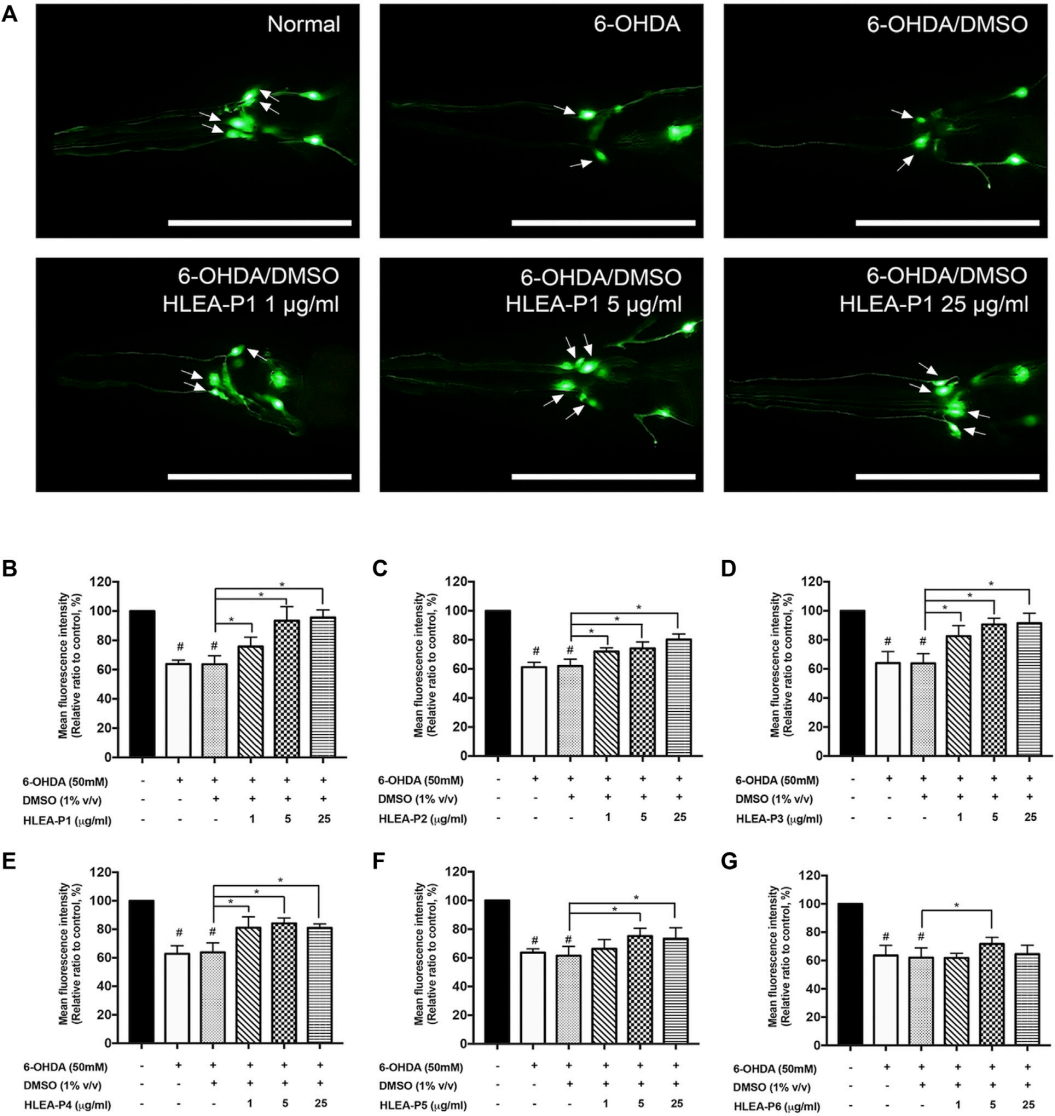
Effect of HLEA compounds 1–6 against 6-OHDA-induced DAergic neurodegeneration in *C. elegans* PD model. **(A)** Representative GFP expression pattern in CEP neurons (white arrows) of normal BY250, 6-OHDA-induced BY250, and 6-OHDA-induced worms with HLEA-P1 treatment at doses of 1, 5, and 25 μg/ml. **(B–G)** Graphical representation for relative fluorescent intensity of GFP expression in CEP neurons of normal BY250, BY250 exposed to 6-OHDA, BY250 exposed to 6-OHDA/DMSO, and BY250 exposed to 6-OHDA with HLEA compound 1–6 at doses of 1, 5, and 25 μg/ml. The data are presented as mean ± SD (*n* = 30, number of animals). The hash (#) indicates a significant difference between normal and 6-OHDA-induced worms (*p* < .05). The asterisk (*) indicates significant differences between untreated group (6-OHDA/DMSO) and HLEAs-treated groups at *p* < .05. Scale bar is 100 μm.

In this study, 6 compounds were isolated from HLEA by CC and TLC and designated as HLEA-P1, HLEA-P2, HLEA-P3, HLEA-P4, HLEA-P5, and HLEA-P6. Firstly, we tested the neuroprotective effects of all isolated compounds against 6-OHDA-induced DAergic neurodegeneration. *C. elegans* BY250 strain with GFP-tagged DAergic neurons was used in this study. CEP neurons of *C. elegans* are the most susceptible to 6-OHDA toxicity when compared to other DAergic neurons (Nass et al., 2002). Therefore, we evaluated the viability of DAergic neurons by measuring the GFP intensity of CEP neurons. Our results showed that the GFP intensity significantly decreased to about 60%–64% (*p* < .05) in either 6-OHDA-treated group or 6-OHDA/DMSO-treated group (Figures 1B–G), suggesting that 6-OHDA-induced DAergic neurodegeneration was successfully established. At particular doses, certain HLEA compounds could restore DAergic neurons from 6-OHDA toxicity as reflected by the increase of GFP intensities in the treated group compared to the untreated group (6-OHDA/DMSO).

HLEA-P1 at 1, 5, and 25 μg/ml significantly restored GFP intensity of CEP neurons to 75.74%, 93.48%, and 95.63% (*p* < .05), respectively, when compared to untreated worms (Figure 1B). HLEA-P2 at doses of 1, 5, and 25 μg/ml significantly restored GFP intensity of CEP neurons in 6-OHDA induced-worms to 71.95%, 74.16%, and 80.28% (*p* < .05), respectively, compared to that of the untreated group (Figure 1C). HLEA-P3 at 1, 5, 25, and 50 μg/ml considerably increased GFP intensity of CEP neurons to 82.50%, 90.56%, and 91.54% (*p* < .05), respectively, when compared to untreated group (Figure 1D). HLEA-P4 at 1, 5, and 25 μg/ml showed significantly recovered GFP intensity of CEP neurons to 81.11%, 84.27%, and 81.41% (*p* < 0.05), respectively (Figure 1E). HLEA-P5 at 5 and 25 μg/ml significantly restored GFP intensity to 75.24% and 73.40% (*p* < .05), respectively, compared to untreated worms (Figure 1F). For HLEA-P6 treatment, only 5 μg/ml of HLEA-P6 slightly increased GFP intensity of CEP neurons to approximately 71.69% (*p* < .05) when compared to untreated group (Figure 1G).

Following these results, the most effective compound that conferred protection to DAergic neurons from 6-OHDA toxicity was HLEA-P1 at concentrations of 5 and 25 μg/ml. Therefore, HLEA-P1 was selected for further experiments to evaluate its anti-Parkinson effects and elucidate mechanisms of action.

### 3.2 Effects of HLEA-P1 on dopamine-dependent behaviors in 6-OHDA-induced *C. elegans* PD model

Investigation of behavioral phenotypes is widely used for detecting DAergic function (Maulik et al., 2017). We therefore investigated whether HLEA-P1 could improve dopamine-dependent behaviors in 6-OHDA-induced worms. Dopamine signal of *C. elegans* is responsible for several behaviors (Maulik et al., 2017). In this study, food sensing response and ethanol avoidance behavior were used as behavioral readouts (Maulik et al., 2017). Food sensing behavior or basal slowing response refers to the decrease in locomotion of worms when the food is available. In the presence of food, normal wild type worms are able to sense bacterial texture and decrease their locomotor rate. In contrast, *C. elegans* PD models with deficit DAergic neurons fail to slow their movement, exhibiting abnormal decreased basal slowing rate (BSR) in the presence of bacteria (Sawin et al., 2000).

**FIGURE 2:**
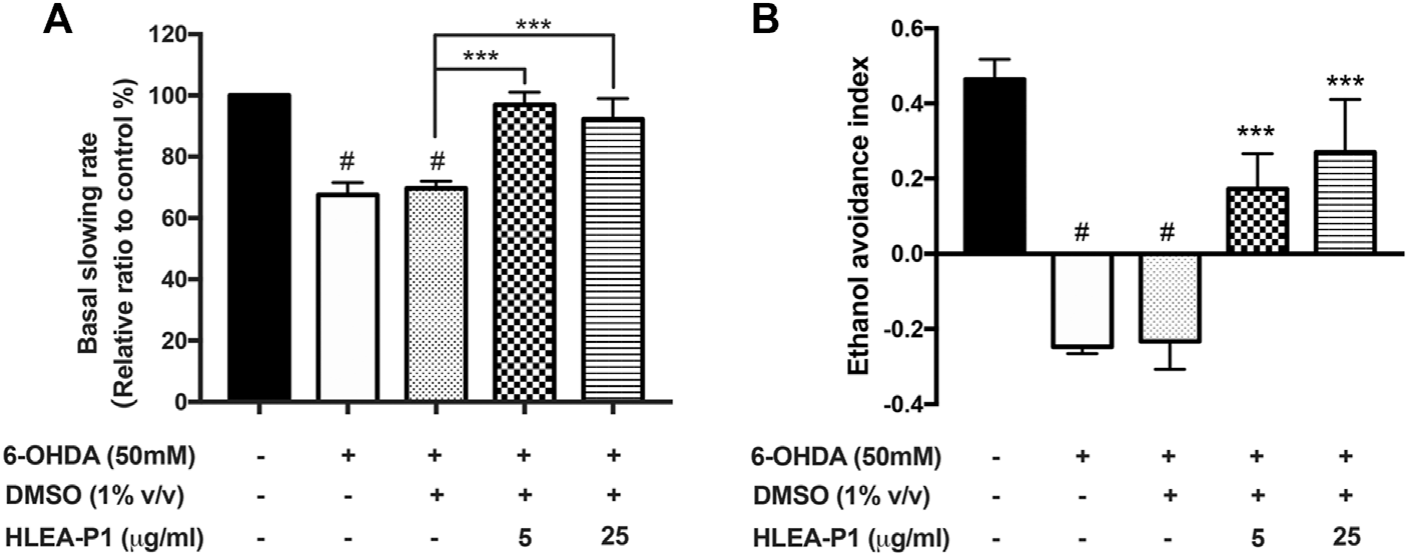
Effect of HLEA-P1 on dopamine dependent behaviors in 6-OHDA-induced *C. elegans* PD model. **(A)** Graphical representation for relative basal slowing rate. The data are presented as mean ± SEM (with three independent replicates, *n* = 30 number of animals per replicate). **(B)** Graphical representation for ethanol avoidance index. The data are presented as mean ± SEM (three independent replicates, *n* ≥ 50 number of animals per replicate). The hash (#) indicates a significant difference between normal and 6-OHDA-induced worms (*p* < .05). The asterisk (*) indicates significant differences between untreated group (6-OHDA/DMSO) and HLEA-P1-treated groups, ***p < .001.

Our results showed that BSR was significantly decreased to about 67.56% and 69.64% in 6-OHDA-induced and 6-OHDA/DMSO-induced worms, respectively (*p* < .05), indicating functional deficit of DAergic neurons in food sensing response. Treatment with HLEA-P1 significantly restored BSR to 96.93% and 92.23% at 5 and 25 μg/ml, respectively, when compared to the untreated group (*p* < .001) (Figure 2A). The loss of dopamine signaling also affect ethanol avoidance behavior in *C. elegans* [19]. Comparing to normal worms, worms treated with 6-OHDA showed significant reduction in ethanol avoidance index. The ethanol avoidance index of normal worms was about 0.46 while it was decreased to −0.25 and −0.23 in 6-OHDA-induced and 6-OHDA/DMSO-induced worms, respectively (*p* < .05). As expected, ethanol avoidance deficit can be improved in HLEA-P1 treatments. The results showed that 5 and 25 μg/ml of HLEA-P1 remarkably increased ethanol avoidance index to 0.17 and 0.27 (*p* < .001), respectively, compared to the untreated group (Figure 2B). Taken together, the results suggested that HLEA-P1 could restore dopamine-dependent behaviors in 6-OHDA induced DAergic neurodegenerative *C. elegans.*

### 3.3 Effect of HLEA-P1 on oxidative stress reduction

**FIGURE 3:**
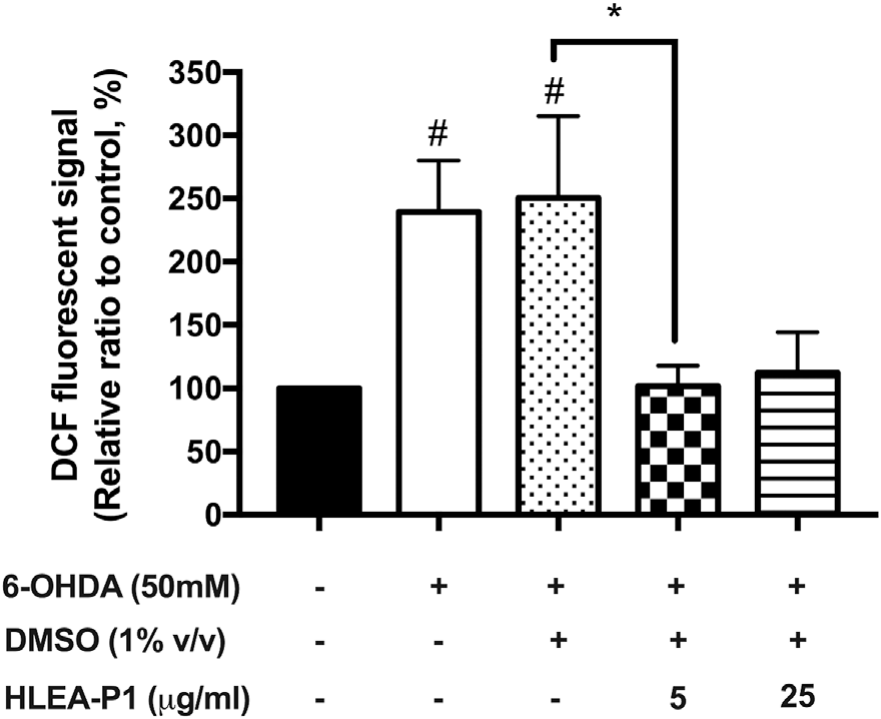
Effect of HLEA-P1 on reactive oxygen species in 6-OHDA-induced *C. elegans* PD model. Graphical representation for DCF fluorescence (%) in normal, 6-OHDA-, 6-OHDA/DMSO-, and 6-OHDA/DMSO plus HLEA-P1-treated worms compared to the untreated group. The data are presented as mean ± SEM (three independent replicates, *n* = 30 number of animals per replicate). The hash (#) indicates a significant difference between untreated and 6-OHDA-, 6-OHDA/DMSO-treated worms. The asterisk (*) indicates significant differences between 6-OHDA-, 6-OHDA/DMSO- and HLEA-P1-treated groups at *p* < .05.

We hypothesized that the mechanism of 6-OHDA in affecting neurotoxicity is oxidative stress. Mechanically, 6-OHDA targets mitochondria and subsequently causes the overproduction of ROS. Therefore, we were interested in whether HLEA-P1 could reduce the oxidative stress to alleviate 6-OHDA toxicity in *C. elegans* model. In the present study, intracellular ROS was measured by H_2_DCF-DA assay. Intracellular ROS, especially H_2_O_2_ can convert non-fluorescence molecules, H_2_DCF-DA, to the fluorescence DCF molecules. Therefore, the ROS level can be interpreted by the DCF fluorescence levels. As shown in Figure 3, when compared to normal worms, intracellular ROS level dramatically increased to 239.58% and 250.60% in 6-OHDA-induced and 6-OHDA/DMSO-induced groups, respectively (*p* < .05). When the worms were treated with HLEA-P1 at 5 μg/ml, intracellular ROS significantly decreased to 101.81% (*p* < .05), the level that was very close to that of the untreated worms. Meanwhile, 25 μg/ml of HLEA-P1 could also significantly reduced ROS to the level that is insignificantly different compared to that of the untreated worms. These results are suggesting that HLEA-P1 at 5 μg/ml is the most effective in the reduction of oxidative stress in 6-OHDA-induced *C. elegans* PD model.

### 3.4 Effect of HLEA-P1 against α-synuclein aggregation and movement deficit in worms expressing α-synuclein

**FIGURE 4:**
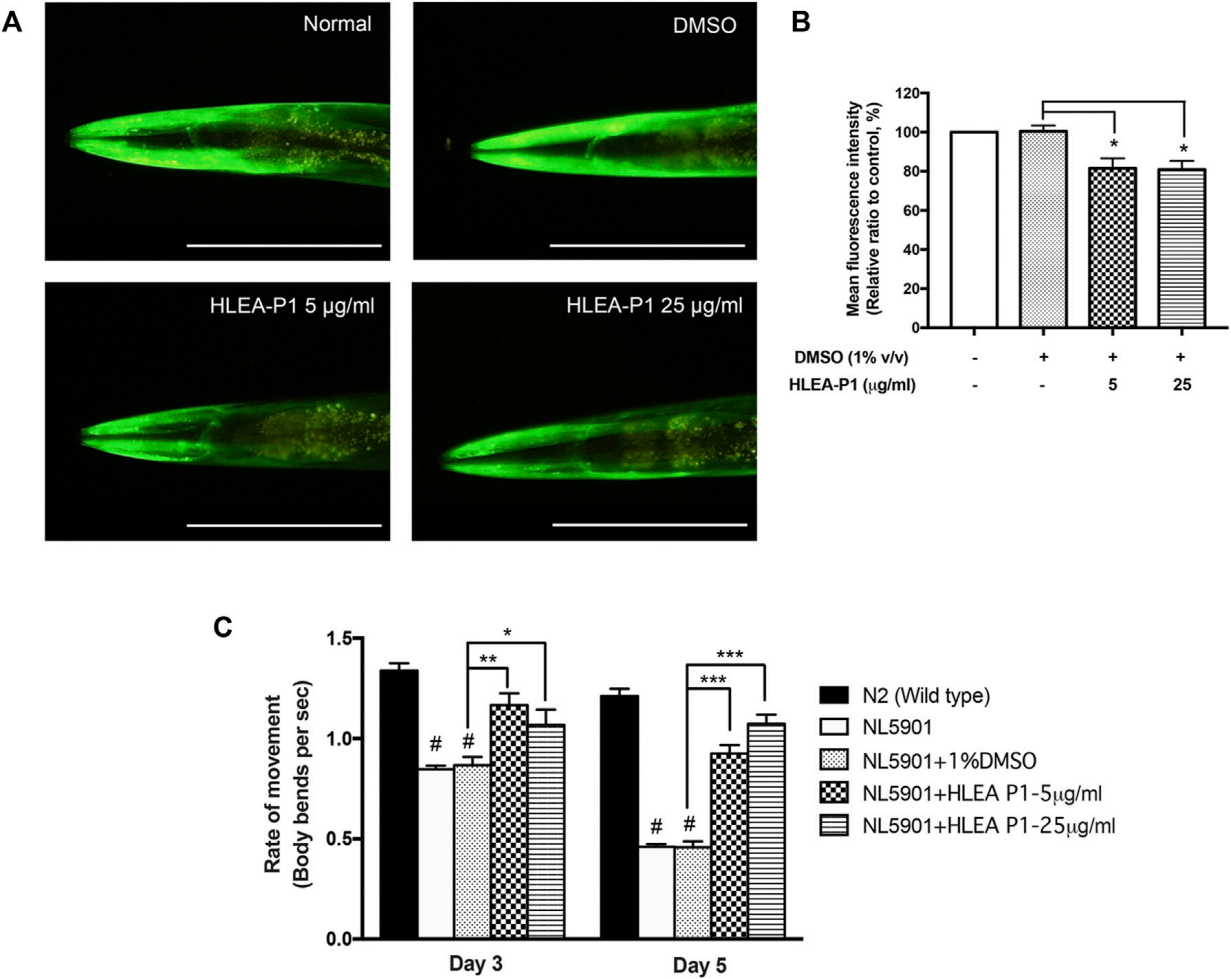
Effect of HLEA-P1 against α-synuclein aggregation and movement deficit in NL5901 *C. elegans* expressing α-synuclein. **(A)** Representative of YFP-tagged α-synuclein expression in body wall muscle cells of NL5901 strain. **(B)** Graphical representation for relative fluorescent intensity of YFP expression. **(C)** Graphical representation of the rate of movement of *C. elegans* with HLEA-P1 treatment. The data are presented as mean ± SD (*n* = 30, number of animals). The hash (#) indicates a significant difference between N2 wild type and NL5901 worms (*p* < .05). The asterisk (*) indicates significant differences between untreated group (DMSO) and HLEA-P1-treated group, *p < .05, **p < .01, ***p < .001. Scale bar is 200 μm.

Next, we determined the effect of HLEA-P1 on α-synucleinopathies, another hallmark of PD. We used the transgenic *C. elegans* NL5901 which expresses the YFP-tagged α-synuclein that can be detectable under a fluorescence microscope. In this study, worms treated with 5 and 25 μg/ml of HLEA-P1 showed significantly decreased YFP intensity to 81.51% and 80.90%, respectively, when compared to untreated group (*p* < .05). In other words, the level of α-synuclein aggregation was significantly diminished by about 18.49% and 19.10% in the transgenic worms treated with 5 and 25 μg/ml of HLEA-P1, respectively (Figures 4A, B).

Many studies reported the association of decline muscular function and protein aggregation in the transgenic *C. elegans* [24]. Previous study showed that NL5901 worms exhibited decreased trashing or swimming behavior with aging when compared with normal wild-type worms (Maulik et al., 2017). To investigate whether HLEA-P1 could improve thrashing deficits, we performed this assay at two time points (adult day 3 and day 5 of *C. elegans*). The results showed that wild-type N2 worms have thrashing rate at 1.34 and 1.21 in day 3 and day 5 of adulthood, respectively. In contrast, day 3 adult NL5901 worms showed significantly decreased thrashing rate to 0.85 when compared to wild-type worms with thrashing rate of 1.34 (*p* < .05) (Figure 4C). Such altered motility was more obvious in day 5 the adult population, which have thrashing rate at 0.46 (*p* < .05). This could be due to age-dependent aggregation of α-synuclein interfering with the worm motility. The results demonstrated that this impairment can be improved upon treatment with HLEA-P1. The rate of movement of day 3 adult treated with 5 and 25 μg/ml of HLEA-P1 were moderately increased to 1.17 and 1.07, respectively (*p* < .01 and *p* < .05, respectively). The significant restorative effect can be observed in day 5 of adulthood in which the rate of movement increased to 1.02 and 0.87 in worms treated with 5 and 25 μg/ml of HLEA-P1, respectively (*p* < .01) (Figure 4C). Overall, these results suggest that HLEA-P1 could improve movement deficits in PD worms which is suppressed by α-synuclein aggregation.

### 3.5 Effect of HLEA-P1 on lipid restoration in transgenic worms expressing α-synuclein

**FIGURE 5:**
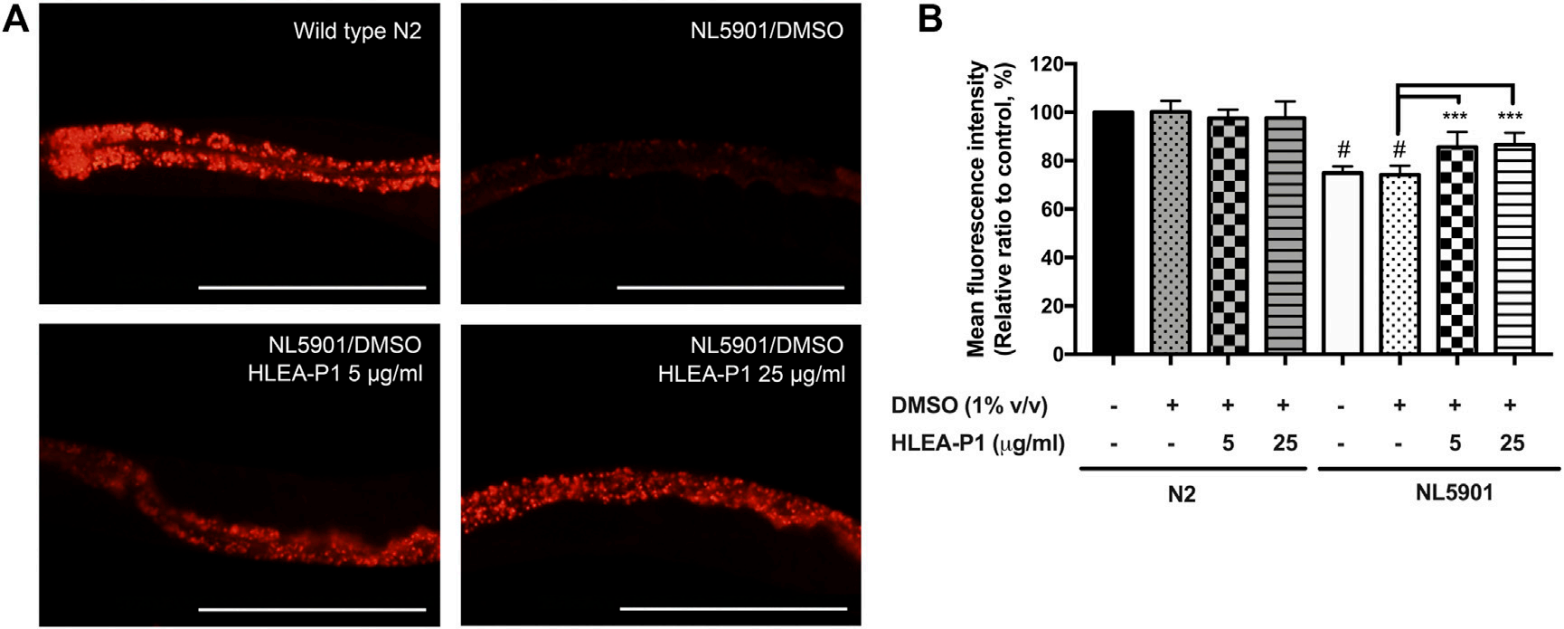
Effect of HLEA-P1 on lipid restoration in transgenic NL5901 worms expressing α-synuclein. **(A)** Representative Nile red fluorescent images of wild-type N2, *C. elegans* NL5901 and *C. elegans* NL5901 treated with HLEA-P1 at 5 and 25 μg/ml. **(B)** Graphical representation for Nile red fluorescent signal. The data are presented as mean ± SEM (with three independent replicates, *n* ≥ 30 number of animals per replicate). The hash (#) indicates a significant difference between wild-type N2 and NL5901 worms (*p* < .05). The asterisk (*) indicates significant difference between untreated group (NL5901/DMSO) and HLEA-P1-treated groups, ***p < .001. Scale bar is 200 μm.

Aggregation of α-synuclein has been reported to cause oxidative degradation of lipids (Maulik et al., 2017). In addition, several studies identified abnormal decrease of the lipid contents in PD worms expressing α-synuclein (Maulik et al., 2017). Therefore, we further investigated the effect of HLEA-P1 on lipid deposition in NL5901 worms using Nile red staining. The results showed that NL5901 worms exhibited 25% lower lipid content than that of wild-type N2 (Figure 5). While HLEA-P1 did not change the lipid deposition in normal wild-type N2, treatment with HLEA-P1 at 5 and 25 μg/ml significantly increased lipid deposition in NL5901 strain to 85.65% and 86.65% of the wild type (*p* < .001), respectively (Figure 5).

### 3.6 Effect of HLEA-P1 on DAF-16 nuclear translocation

**FIGURE 6:**
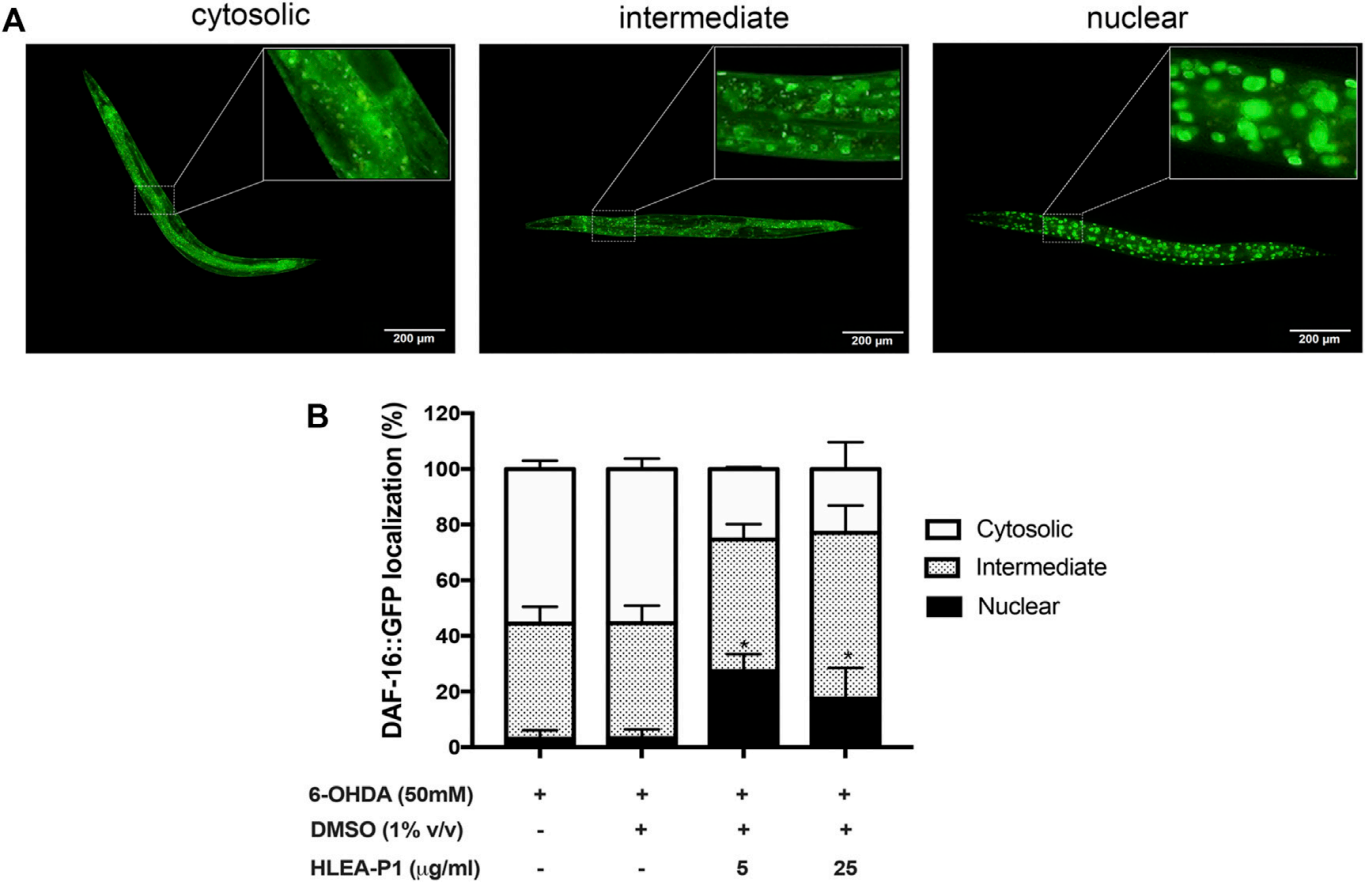
Effect of HLEA-P1 on nuclear localization of transcription factor DAF-16 in 6-OHDA-induced *C. elegans.* **(A)** Representative fluorescent images of different subcellular localization of DAF-16. **(B)** Graphical representation of subcellular distribution of *daf-16*. The data are presented as mean ± SEM (with three independent replicates, *n* ≥ 30 number of animals per replicate). The asterisk (*) indicated significant differences of DAF-16 nuclear localization between untreated group (6-OHDA/DMSO) and HLEA-P1-treated groups at *p* < .05.

Considering the fact that 6-OHDA and α-synuclein aggregation contribute to oxidative stress in DAergic neurons, we hypothesized that HLEA-P1 might be involved in oxidative stress reduction. In *C. elegans*, DAF-16 is one of the major transcription factors of the insulin/IGF-1 signaling (IIS) pathway that provides oxidative stress resistance by gaining access into the nucleus and activating the expressions of antioxidant enzymes (Altintas et al., 2016). Therefore, we investigated the effect of HLEA-P1 on DAF-16 nuclear translocation in TJ356 carrying GFP-tagged DAF-16 protein. At basal condition, DAF-16 localizes in cytoplasm. Under stress, DAF-16 undergoes nuclear translocation to activate transcription of downstream-target genes (Altintas et al., 2016). In this study, subcellular localizations of DAF-16 were classified as cytosolic, intermediate, and nuclear localization as shown in Figure 6A. In normal TJ356, DAF-16 was shown to localize in cytosol, intermediate and nucleus at 88.89%, 8.33%, and 2.78%, respectively. In PD models, 55.58% and 55.37% of 6-OHDA-induced and 6-OHDA/DMSO-induced worms exhibited cytosolic DAF-16 localization. Approximately 41.39% of the former and 41.43% of the latter showed intermediate DAF-16 localization, while only 3.04% and 3.20% exhibited DAF-16 nuclear localization. Treatment with HLEA-P1 at 5 and 25 μg/ml significantly increased DAF-16 nuclear localization up to 27.35% and 17.49%, respectively (*p* < .05) (Figure 6). At the same time, cytosolic DAF-16 localization of 5 and 25 μg/ml treatment was significantly reduced to 25.36% and 22.95%, respectively (*p* < .05). These results suggested that HLEA-P1 triggered DAF-16 translocation from cytoplasm to nucleus in 6-OHDA-induced *C. elegans* PD model.

### 3.7 Effect of HLEA-P1 on the expression of SOD-3 and GST-4

**FIGURE 7:**
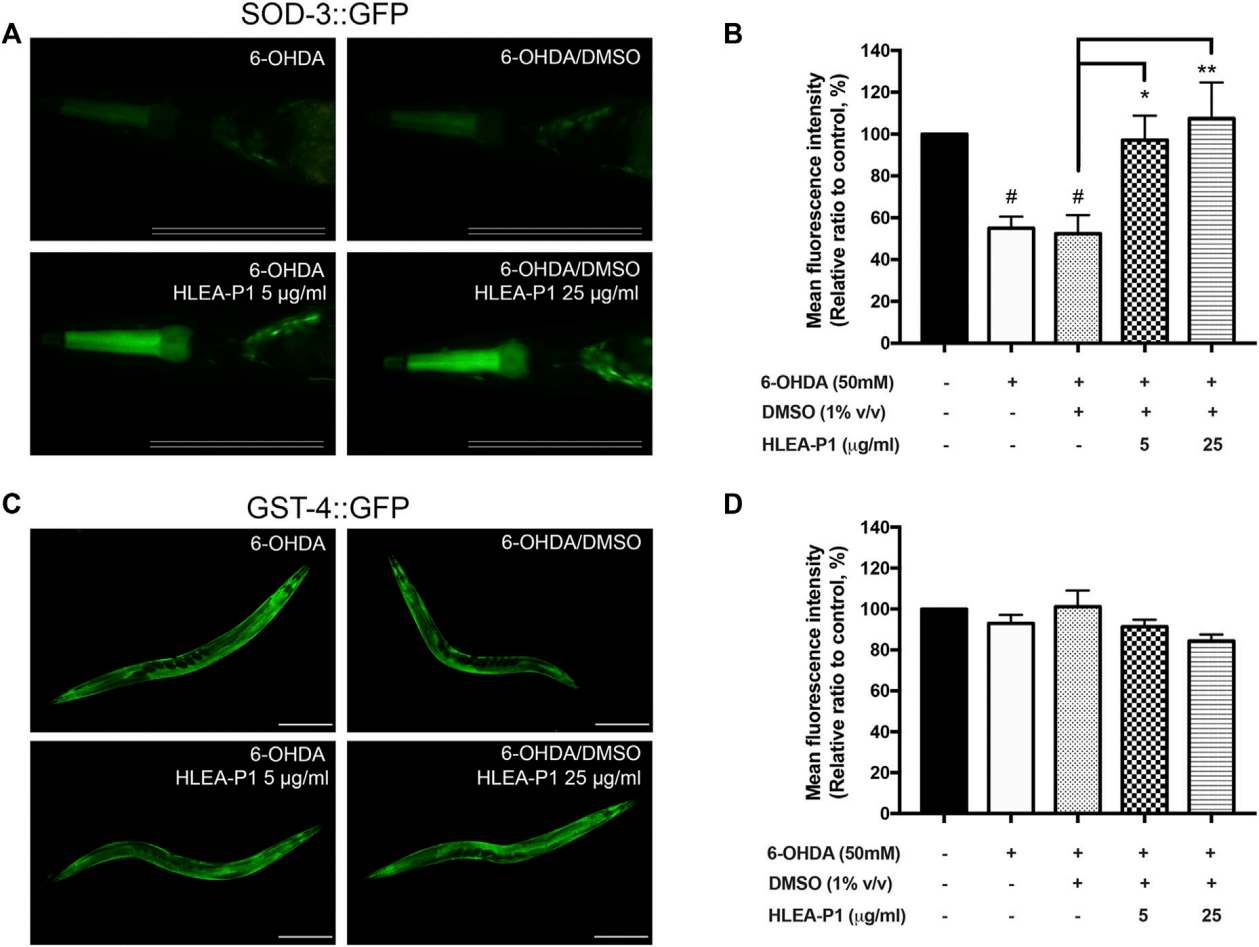
Effect of HLEA-P1 on SOD-3 and GST-4 in *C. elegans* PD model. **(A,B)** Representative SOD-3:GFP expression and mean fluorescent intensity of SOD-3:GFP in transgenic CF1553 in different conditions (normal CF1553, 6-OHDA-induced CF1553, 6-OHDA/DMSO-treated CF1553, and 6-OHDA-induced CF1553 treated with HLEA-P1 at 5 and 25 μg/ml). Scale bar is 100 μm. **(C,D)** Representative GST-4:GFP expression and mean fluorescent intensity of GST-4:GFP in transgenic CL2166 in different conditions (normal CL2166, 6-OHDA-induced CL2166, 6-OHDA/DMSO-treated CL2166 and 6-OHDA-induced CL2166 treated with HLEA-P1 at 5 and 25 μg/ml). Scale bar is 200 μm. The hash (#) indicates a significant difference between wild type N2 and transgenic worms (*p* < .05). The asterisk (*) indicates significant difference between untreated group (6-OHDA/DMSO) and HLEA-P1 treated groups, ***p < .001.

In IIS pathway of *C. elegans*, SKN-1 is another transcription factor regulating oxidative stress resistance. SKN-1 activates the transcription of the anti-oxidant gene such as *gst-4* (*glutathione S-transferase-4*) whereas DAF-16 activates *sod-3* (*superoxide dismutase-3*). Hence, we further confirmed that HLEA-P1 activates DAF-16 but not SKN-1 by investigating its effect on their downstream targets, SOD-3 and GST-4 proteins, using the transgenic strains CF1553 and CL2166, respectively. The results demonstrated that the 6-OHDA-induced and the 6-OHDA/DMSO-induced CF1553 exhibited significantly reduced SOD-3:GFP expression to 55.00% and 52.40%, when compared with normal CF1553 worms. These data indicated the downregulation of SOD-3:GFP upon the 6-OHDA treatment. As expected, treatment with HLEA-P1 at 5 and 25 μg/ml remarkably increased SOD-3 to 97.15% and 107.42%, respectively (*p* < .05 and *p* < .01, respectively) when compared with the untreated group (Figures 7A, B). While, 6-OHDA-induced and 6-OHDA/DMSO-induced CL2166 did not show significant difference in GST-4 expression when compared with the normal CL2166 worms. Moreover, treatment with HLEA-P1 at 5 and 25 μg/ml could not enhance the GST-4:GFP expression in CL2166 (Figures 7C, D). Taken together, these results suggested that treatment with HLEA-P1 significantly activates expression of SOD-3, a downstream target of DAF-16, rather than GST-4 which is a downstream target of SKN-1, in 6-OHDA-induced *C. elegans*.

### 3.8 Effect of HLEA-P1 on mRNA expressions of DAF-16 target genes

**FIGURE 8:**
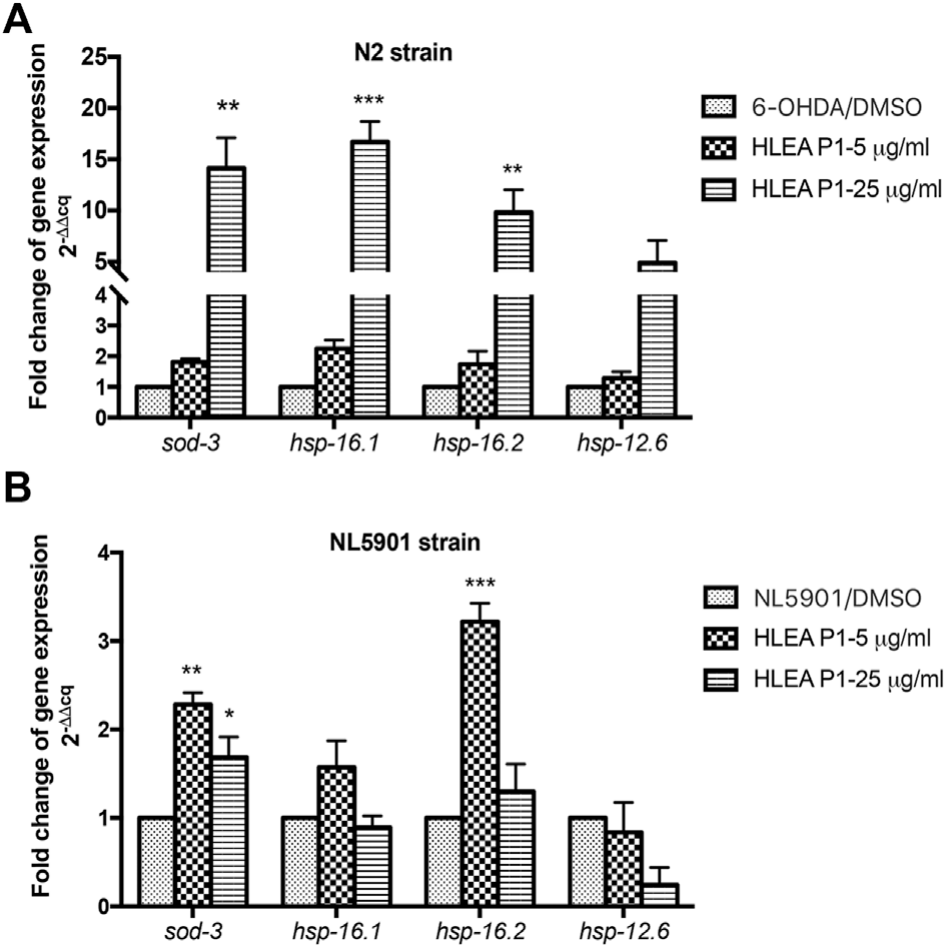
Effect of HLEA-P1 on expressions of mRNAs of DAF-16 target genes in *C. elegans* PD models. Graphical representation for fold change of gene expression level (2^−(ΔΔCq)^) of DAF-16 target genes in 6-OHDA-induced worms **(A)** and NL5901 worms overexpressing α-synuclein **(B)**. The relative mRNA expressions were normalized with internal control *act-1*. The data represented as a mean ± SEM (three independent replicates, *n* = 800–1,000 number of animals per replicate). The asterisk (*) indicates significant differences between untreated group (DMSO) and HLEA-P1-treated group, *p < .05, **p < .01, ***p < .001.

Next, we investigated the mRNA expression of DAF-16 target genes including *sod-3*, *hsp-16.1*, *hsp-16.2*, and *hsp-12.6* in 6-OHDA-induced *C. elegans* model and transgenic *C. elegans* NL5901 expressing α-synuclein model. *sod-3* is an anti-oxidative gene that upregulated by IIS dependent DAF-16 transcription factor (Tullet et al., 2008). *hsp-16.1*, *hsp-16.2*, and *hsp-12.6* are small heat shock protein, HSP27 homologs, involve in stress resistance (Hsu et al., 2003). Downregulation of hsp27 is observed in several neurodegenerative diseases including PD while overexpression of hsp27 showed neuroprotective effects against neurodegenerative diseases (Vidyasagar et al., 2012; Venugopal et al., 2019). In 6-OHDA-induced model, the results showed that treatment with HLEA-P1 at 5 μg/ml insignificantly upregulated expressions of mRNAs for *sod-3* (1.8-fold), *hsp-16.1* (2.25-fold), *hsp-16.2* (1.74-fold), and *hsp-12.6* (1.29-fold) when compared to untreated group (*p* > .05) Figure 8A. In contrast, 25 μg/ml HLEA-P1 significantly upregulated expressions of mRNAs for *sod-3* (14.16-fold), *hsp-16.1* (16.71-fold), and *hsp-16.2* (9.84-fold), compared to untreated group (*p* < .05). Considerable upregulation with no significant difference of *hsp-12.6* mRNA (4.91-fold) was also observed in 25 μg/ml HLEA-P1 treatment. Despite insignificant upregulation by treatment at low concentration, our results showed that HLEA-P1 could upregulate *daf-16* downstream target-genes including *sod-3*, *hsp-16.1*, and *hsp-16.2*. In NL5901 model, treatment with 5 and 25 μg/ml HLEA-P1 significantly upregulated expression *sod-3* mRNA by 2.28-fold and 1.69-fold, respectively (*p* < .05) when compared to untreated group Figure 8B. Moreover, treatment with 5 μg/ml HLEA-P1 significantly increased expression of *hsp-16.2* mRNA compared to untreated group (*p* < .05). These results suggested that 5 μg/ml HLEA-P1 significantly upregulated *sod-3* and *hsp-16.2*, but not *hsp16.1* and *hsp12.6.*

### 3.9 Chemical characterization of compounds 1-6 from *H. leucospilota* EtOAc fraction

**FIGURE 9:**
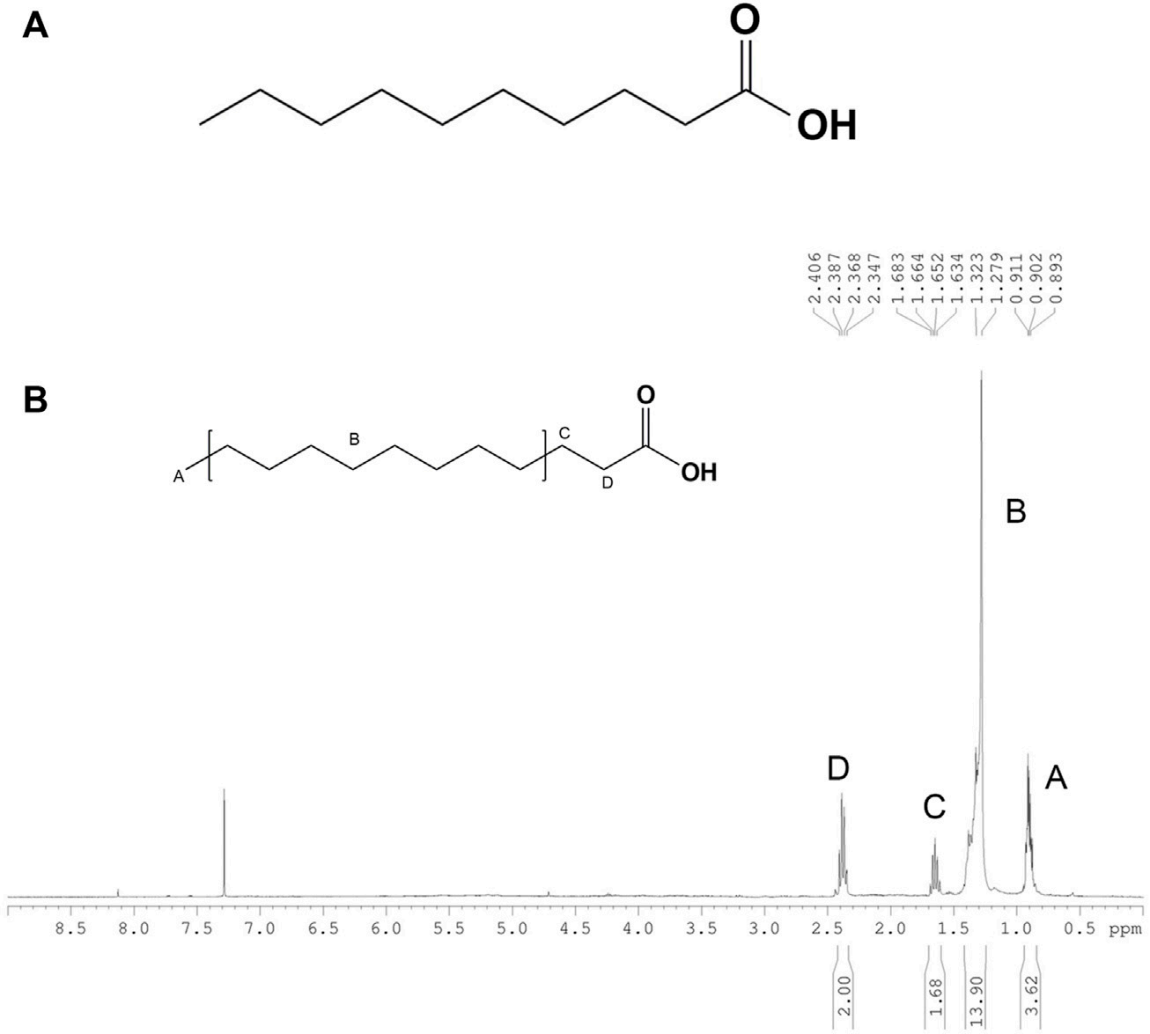
**(A)** Chemical structure of HLEA-P1. **(B)** ^1^H-NMR spectrum of HLEA-P1, decanoic acid or capric acid in CDCl_3_.

Based on structural elucidation by ^1^H and ^13^C NMR analysis, purified compounds 1-6 in EtOAc fraction were identified and shown in Supplementary Material (Supplementary Figure S1). For HLEA-P1, the ^1^H-NMR/^13^C NMR spectra showed that white powder, HR-TOFMS (ES^+^): m/z 195.1062 [M + Na]^+^, calcd for C_10_H_20_O_2_+Na. ^1^H-NMR (CDCl_3_, 400 MHz): 0.85 (3H, t = 7.2, H-10), 1.24 (10H, m, H-5-9), 1.60 (2H, m, H-3), 2.31 (2H, t, H-2). ^13^C-NMR (CDCl_3_, 100 MHz): 14.1 (C-10), 22.7 (C-9), 24.7 (C-3), 29.0 (C-4), 29.3 C-5, 7), 29.6 (Cδ 6), 31.9 (C-8), 34.0 (C-2), 178.4 (COOH). According to a combination of ^1^H-NMR/^13^C NMR spectra, HLEA-P1 was identified as Decanoic acid (Capric acid), C_10_H_20_O_2_ (Figure 9A and Supplementary Figures S2, S3). As shown in Figure 9B, Peak A at 0.85 ppm indicates a presence of terminal methyl group (CH_3_) bounded to the C_9_. Peak B at 1.24 ppm displayed a long chain of methylene protons (CH_2_) of the C_4_-C_9_ atoms. Peak C at 1.60 ppm is related to 2 protons bound to the C_3_ atom. Peak D at 2.31 ppm is corresponded with methylene protons (CH_2_) of C_2_.

## 4 Discussion

In our recent report (Malaiwong et al., 2019), the crude extract of *H. leucospilota* ethyl acetate fraction (HLEA) exhibited anti-Parkinson’s activities in *C. elegans* PD models. In this ensuing study, we demonstrated that such HLEA fraction consists of 6 bioactive compounds (Supplementary Figures S1–S12) with HLEA-P1 showing the most pronounced effect against 6-OHDA-induced DAergic neurodegeneration (Figure 1). Hence, our present study focused on the anti-Parkinson’s effects of HLEA-P1 and its mechanism of action at molecular level. Firstly, we found that HLEA-P1 (at 5 and 25 μg/ml) could restore DAergic neurons from 6-OHDA-induced neurodegeneration in the BY250 strain (Figure 1). Consistent with the restored viability of the DAergic neurons, HLEA-P1 improved food sensing and ethanol avoidance behaviors in 6-OHDA-induced worms (Figure 2). In addition to the DAergic neurodegeneration model, we also tested the effect of HLEA-P1 on the α-synucleinopathies using the transgenic *C. elegans* NL5901 which over-expresses human α-synuclein. HLEA-P1 (at 5 and 25 μg/ml) significantly decreased α-synuclein aggregation which was correlated with the improvement of thrashing behavior in NL5901 worms (Figure 4).

Regardless of the exogenous and the endogenous sources, oxidative stress is considered to be the common molecular event in PD pathogenesis that triggers neurodegeneration (Chakraborty et al., 2013). The 6-OHDA is a neurotoxin which mechanistically causes mitochondrial impairment leading to overproduction of the intracellular ROS (Blum et al., 2001). The α-synuclein contributes to oxidative stress by inhibiting mitochondrial complex I activity, decreasing mitochondrial function, and generating the excessive ROS level (Scarlata and Golebiewska, 2014). We therefore investigated whether HLEA-P1 could reduce oxidative stress, thereby alleviating Parkinsonism in *C. elegans* PD models. We measured intracellular ROS using H_2_DCF-DA assay and found that HLEA-P1, in particular 5 μg/ml, significantly reduced intracellular ROS level in 6-OHDA-induced worms (Figure 3). It has been well known that the overproduction of the intracellular ROS causes damage to the biomolecules such as proteins, DNA, and lipids (Chakraborty et al., 2013). The aggregation of α-synuclein contributes to oxidative degradation of lipids (Angelova et al., 2015). Although we did not measure intracellular ROS in NL5901 overexpressing human α-synuclein model, we investigated the lipid accumulation in this strain using Nile red staining. The results revealed that HLEA-P1 had no significant effect on lipid accumulation in wild-type worms, but could restore lipid content in transgenic worms expressing α-synuclein (Figure 5). These results implied that HLEA-P1 might not act directly on lipid metabolism but rather indirectly through its reductive effects on oxidative stress and α-synuclein aggregation.

The IIS pathway is crucial for regulating neuronal physiology including neuronal development, synaptic maintenance, neuronal growth, and survival (Bassil et al., 2014). Growing evidence indicated the alteration of this pathway in many neurodegenerative diseases including PD (Bassil et al., 2014; Bassil et al., 2022). Meanwhile, targeting IIS pathway showed positive benefits for disease modification (Bassil et al., 2014); for instance, knock down of DAF-2 (an orthologue of mammal insulin/IGF receptor) decreased α-synuclein aggregation in *C. elegans* model (Haque et al., 2020). Interestingly, several bioactive compounds from sea cucumbers could modulate the IIS pathway, for example, the crude extract from *H. scabra* could exert anti-oxidant and anti-aging properties (Jattujan et al., 2018). Another study by Kitisin et al. (2019) reported that the extract of *H. leucospilota* body wall promoted the lifespan extension and stress tolerance in *C. elegans* model by targeting DAF-16 transcription factor of IIS pathway. Therefore, in this study we explored whether HLEA-P1 exerted anti-PD activities through IIS/DAF-16 pathway. Our results showed that HLEA-P1 activates DAF-16 nuclear localization in 6-OHDA-induced worms (Figure 6). This result was corroborated by a significant increase in SOD-3:GFP expression in the 6-OHDA-induced CF1553 treated with HLEA-P1 while expression of GST-4, a downstream target of SKN-1, was not significant (Figure 7). Therefore, it could be concluded that HLEA-P1 promotes stress resistance by activating DAF-16 transcription factor of IIS pathway rather than SKN-1 transcription factor.

To further confirm that anti-Parkinson’s activities of HLEA-P1 is mediated by de-activating IIS pathway which, in turn, allows DAF-16 to enter the nucleus and activate its target genes, we measured the expressions of mRNAs of *sod-3*, *hsp-16.1*, *hsp-16.2*, and *hsp-12.6* in *C*. *elegans* PD models. Our results showed that expression levels of mRNAs of *sod-3* and *hsp27* homologs (*hsp-16.1*, *hsp-16.2*, and *hsp-12.6*) were all upregulated in the 6-OHDA-induced worms treated with HLEA-P1. Recently, several lines of evidence indicated that the HSPs can interact not only with misfolded proteins to prevent unfolded protein response (UPR) but also with native proteins to modulate several normal cellular functions (Venugopal et al., 2019). During stress conditions, smHSP27 acts as the anti-oxidant, scavenging intracellular ROS by increasing glutathione level (Venugopal et al., 2019). Moreover, HSP27 can act as an anti-apoptotic agent by inhibiting mitochondrial dysfunction and apoptotic pathway (Venugopal et al., 2019). Previously, it was shown that overexpression of *hsp27* exhibited protective effects against 6-OHDA-induced toxicity by inhibiting cytochrome C release, caspase activation, thereby apoptosis (Gorman et al., 2005). Hence, we hypothesized that upregulation of *sod-3* and HSP27 homologs by HLEA-P1 may enhance anti-oxidative and anti-apoptotic capacities, rendering the 6-OHDA less toxic.

In our present study we demonstrated that in *C. elegans* NL5901, HLEA-P1 (5 and 25 μg/ml) significantly upregulated transcription level of *sod-3*. Similarly, a previous study reported that upregulation of *sod-3* by dianxianning, an anti-epileptic drug, reduced Aβ oligomers and its toxicity in *C. elegans* AD model (Zhi et al., 2017). An *in vitro* study showed that the overexpression of SOD-3 protected SH-SY5Y cell against Aβ-induced-toxicity by inhibiting ROS generation by the mitochondrial pathway (Yang et al., 2009). Deletion of *sod-3* aggravated impaired motility of transgenic Aβ worm (Minniti et al., 2015). Based on PD pathogenesis, α-synuclein can interact with mitochondria complex I, leading to dysfunctional mitochondria and oxidative stress (Hsu et al., 2000). In turn, the excessive ROS causes the aggregation of α-synuclein and acceleration of neuronal cell death (Scarlata and Golebiewska, 2014). We, therefore, proposed that HLEA-P1 may increase anti-oxidative capacity through the upregulation of *sod-3,* thereby decreasing α-synuclein aggregation and its toxicity. *C. elegans* NL5901 treatment with low dose of HLEA-P1 (5 μg/ml) showed a significant upregulation of *hsp* including *hsp16.1* and *hsp16.2.* The activation of *hsp16.1* and *hsp16.2* might promote the clearance system of α-synuclein to reduce α-synuclein aggregation in NL5901. Unlike other *hsps*, *hsp-12.6* was not upregulated by 5 μg/ml HLEA-P1 treatment. When compared to other HSPs, *hsp12.6* contains unusually short N and C-terminal regions which affects its chaperone activity (Leroux et al., 1997). Therefore, we did not observe upregulation of mRNA level of *hsp12.6* in NL5901 model with proteotoxic stress, while upregulation of *hsp-12.6* that might confer anti-oxidative and anti-apoptotic capacity was observed in the 6-OHDA-induced model. Overall, our results demonstrated that a low dose of HLEA-P1 (5 μg/ml) suppressed α-synuclein aggregation and its toxicity by upregulation of *sod-3*, *hsp16.1*, and *hsp16.2* in NL5901. However, 25 μg/ml of HLEA-P1 did not upregulate mRNA expression of *hsp*, indicating that protective effects against α-synuclein aggregation of high concentration may lead to unresponsive genes or involve other pathways. To further clarify other possible targets of HLEA-P1, in the future study a transcriptomic analysis can be performed to verify the upregulated and downregulated genes that may be involved, then the related mechanisms of those genes can further be investigated through molecular and protein analysis methods.

Chemical structure elucidation demonstrated that HLEA-P1 is a medium chain fatty acid (MCFA) with 10 carbon atoms named decanoic acid or capric acid. It is well established that this sea cucumber contains high essential fatty acids (Roopashree et al., 2021). Typically, decanoic acid and other MCFAs have been widely used as nutritional therapy (Zulkanain et al., 2020). MCFA have been shown to exhibit anti-Alzheimer’s, anti-inflammation, as well as anti-oxidative effects (Zulkanain et al., 2020). Here, we reported a reduction of intracellular ROS and upregulation of *sod-3* by decanoic acid in *C. elegans* PD model. Neuroprotection by MCFA has been reported in the AD model. Reduced oxidative stress and increased survival of neurons were observed in amyloid beta-exposed cortical neurons treated with coconut oil containing decanoic acid (Nafar and Mearow, 2014). Moreover, decanoic acid-enriched oil has been reported to increase activities of antioxidative enzymes CAT, SOD, GSH and GPx, *in vivo* (Sengupta and Ghosh, 2012). In intestinal cell, decanoic acid suppressed cyclophosphophamide-induced oxidative stress through activating enzyme activity of SOD and GPX and their gene expression (Lee and Kang, 2017). Previous study also reported that decanoic acid reduces oxidative stress in neuroblastoma cells by activating enzyme activity of catalase but not its transcriptional level (Mett and Müller, 2021). However, activity of SOD was not upregulated by decanoic acid treatment (Nafar and Mearow, 2014). These lines of evidence support our hypothesis that decanoic acid isolated from *H. leucospilota* alleviates 6-OHDA toxicities by upregulating antioxidative defense in *C. elegans* PD model. Moreover, previous study reported that decanoic acid activates autophagy by upregulating autophagy-inducing protein including *Atg1* and *Atg8* (Warren et al., 2021). Autophagy is the major clearance system of α-synuclein aggregation (Maiti et al., 2017). It is possible that high dose of decanoic acid might reduce α-synuclein aggregation and its toxicity by targeting autophagy process. Effect of decanoic acid on reducing lipid peroxidation has also been reported (Sengupta and Ghosh, 2012; Lee and Kang, 2017). Previous study showed decreasing of malondialdehyde (MDA), a marker of lipid peroxidation, following treatment with decanoic acid-enrich oil (Sengupta and Ghosh, 2012; Lee and Kang, 2017; Yoon et al., 2018). In this study, we found recovered lipid deposition in *C. elegans* PD model treated with decanoic acid. Antioxidative enzymes such as SOD and GPx play role in controlling lipid peroxidation (Devi et al., 2000). Thus, the restoration of lipid deposition we observed might be the consequence of antioxidative activity of decanoic acid.

Taken together, we found that anti-Parkinson’s potential of ethyl acetate fraction of the crude extract of the *H. leucospilota* body wall, is attributable to decanoic acid. Our findings substantiated nutrition property of the sea cucumber, *H. leucospilota,* for use as a functional food and nutraceuticals for PD and possibly also other neurodegenerative diseases of similar etiology. However, to promote *H. leucospilota*-derived decanoic acid as a nutraceutical for humans, its anti-PD effects, toxicity, and underlying molecular mechanism of action need to be verified in higher mammal models such as mouse. Moreover, comparison among decanoic acid isolated from *H. leucospilota* with those isolated from other organisms or its synthetic forms should also be studied for developing the most economical and efficient MCFA as a nutritional therapy against Parkinson’s disease.

## 5 Conclusion

In this study, we reported that decanoic acid isolated from ethyl acetate fractions of *H. leucospilota* body wall could attenuate DAergic neurodegeneration, reduce α-synuclein aggregation, and improve associated behavioral deficits in *C. elegans* PD models. We found that *H. leucospilota*-derived decanoic acid activated DAF-16 transcription factor which upregulated its target genes including the anti-oxidants genes (such as *sod-3*) and the small heat shock proteins (such as *hsp16.1*, *hsp16.2*, and *hsp12.6*) to enhance oxidative stress and proteotoxic stress resistances in *C. elegans* PD models. Our study revealed for the first time about anti-Parkinson’s effects of decanoic acid-derived from sea cucumbers. Mechanistically, we found that decanoic acid exerts anti-Parkinson’s effects by de-activating IIS pathway and allows DAF16 to enter the nucleus to activate its target anti-oxidation genes and heat shock proteins in *C. elegans* PD models.

## Data Availability

The original contributions presented in the study are included in the article/Supplementary Materials, further inquiries can be directed to the corresponding author.
