# Supplementary material for: Neuroprotective effects of a medium chain fatty acid, decanoic acid, isolated from H. leucospilota against Parkinsonism in C. elegans PD model
Source: Front Pharmacol. 2022 Dec 13;13:1004568. doi: 10.3389/fphar.2022.1004568 (PMC9792845; doi:10.3389/fphar.2022.1004568)
Supplement: Supplementary file 1 [file DataSheet1.DOCX]

***Supplementary data for Manuscript***

***“Neuroprotective effects of a medium chain fatty acid, decanoic acid, isolated from H. leucospilota against Parkinsonism in C. elegans PD model”***

# Supplementary Data

**Compound HLEA-P1 (Capric acid)**

White powder, HR-TOFMS (ES^+^): m/z 195.1062 [M+Na]^+^, calcd for C_10_H_20_O_2_+Na.

^1^H-NMR (CDCl_3_, 400 MHz): 0.85 (3H, t = 7.2, H-10), 1.24 (10H, m, H-5-9), 1.60 (2H, m, H-3), 2.31 (2H, t, H-2).

^13^C-NMR (CDCl_3_, 100 MHz): 14.1 (C-10), 22.7 (C-9), 24.7 (C-3), 29.0 (C-4), 29.3 C-5, 7), 29.6 (C 6), 31.9 (C-8), 34.0 (C-2), 178.4 (COOH).

**Compound HLEA-P2 (Lauric acid)**

White powder, HR-TOFMS (ES^+^): m/z 223.0322 [M+Na]^+^, calcd for C_12_H_24_O_2_+Na.

^1^H-NMR (CDCl_3_, 400 MHz): 0.84 (3H, t = 7.2, H-12), 1.25 (12H, m, H-5,7-11), 1.28 (4H, m, H-4, 6), 1.61 (2H, m, H-3), 2.32 (2H, t, H-2).

^13^C-NMR (CDCl_3_, 100 MHz): 14.1 (C-12), 22.7 (C-11), 24.7 (C-3), 29.0 (C-4), 29.3 C-5, 9), 29.6 (C 6-7), 31.9 (C-10), 34.0 (C-2), 178.4 (COOH).

**Compound HLEA-P3 (Pamitic acid)**

White powder, HR-TOFMS (ES^+^): m/z 279.0796 [M+Na]^+^, calcd for C_16_H_32_O_2_+Na.

^1^H-NMR (CDCl_3_, 400 MHz): 0.86 (3H, t = 7.2, H-16), 1.23 (24H, m, H-4-15), 1.61 (2H, m, H-3), 2.32 (2H, t, H-2).

^13^C-NMR (CDCl_3_, 100 MHz): 14.1 (C-16), 22.7 (C-15), 24.7 (C-3), 29.0 (C-4), 29.3 C-5, 13), 29.6 (C 6-12), 31.9 (C-14), 34.0 (C-2), 178.4 (COOH).

**Compound HLEA-P4 (Linoleic acid)**

White powder; HR-TOFMS (ES^+^): m/z 303.0761 [M+Na]^+^, calcd for C_18_H_32_O_2_+Na.

^1^H-NMR (CDCl_3_, 400 MHz): 0.85 (3H, m, H-18), 1.23 (14H, m, H-4-7, 15-17), 1.61 (2H, m, H-3), 1.98 (6H, m, H-2, 8, 14), 2.32 (2H, t = 7.6, H-11), 5.33 (4H, m, H-9-10, 12-13)

^13^C-NMR (CDCl_3_, 100 MHz): 14.1 (C-18), 22.8 (C-17), 25.0 (C-3), 25.6 (C-11), 27.8 (C-8, 14), 29.0 (C-4), 29.4 (C-5), 29.7 (C-6), 27.8 (C-8, 14), 29.6 (C-15), 29.9 (C-7), 31.9 (C-16), 33.6 (C-2), 128.8 (C-10, 12), 130.3 (C-9, 13), 179.4 (COOH).

**Compound HLEA-P5 (Linoleic Methyl Ester)**

Colorless oil; HR-TOFMS (ES^+^): m/z 317.0760 [M+Na]^+^, calcd for C_19_H_34_O_2_+Na.

^1^H-NMR (CDCl_3_, 400 MHz): 0.85 (3H, m, H-18), 1.28 (14H, m, H-4-7, 15-17), 1.66 (2H, m, H-3), 2.16 (4H, m, H-8, 14), 2.32 (2H, m, H2), 2.80 (2H, m, H-11), 3.61 (3H, s, OCH_3_), 5.33 (4H, m, H-9, 10, 12,13)

^13^C-NMR (CDCl_3_, 100 MHz): 14.1 (C-18), 22.7 (C-17), 25.0 (C-3), 25.6 (C-11), 27.8 (C-8, 14), 29.0 (C-4), 29.4 (C-5), 29.6 (C-15), 29.7 (C-6), 29.9 (C-7), 31.9 (C-16), 51.9 (OCH_3_), 127.3 (C-12,13), 130.3 (C-9, 10), 178.1 (COOH).

**Compound HLEA-P6 (α-Linolenic acid)**

Colorless oil; HR-TOFMS (ES^+^): m/z 301.0761 [M+Na]^+^, calcd for C_18_H_30_O_2_+Na.

^1^H-NMR (CDCl_3_, 400 MHz): 0.89 (3H, m, H-18), 1.30 (8H, m, H-4-7), 1.72 (2H, m, H-3), 2.05 (2H, m, H- 17), 2.40 (4H, m, H-2, 8), 2.84 (4H, m, H-11, 14), 5.43 (6H, m, H-9, 10, 12,13, 15, 16)

^13^C-NMR (CDCl_3_, 100 MHz): 14.3 (C-18), 22.7 (C-3), 24.7 (C-3), 27.8 (C-8), 29.0 (C-4), 29.4 (C-5), 29.7 (C-6), 29.9 (C-7), 25.7 (C-11), 26.6 (C-17), 31.7 (C-14), 34.0 (C-2), 127.3 (C-10), 128.0 (C-15), 128.8 (C-12, 13), 130.3 (C-9, 16), 178.4 (COOH).

# Supplementary Figures


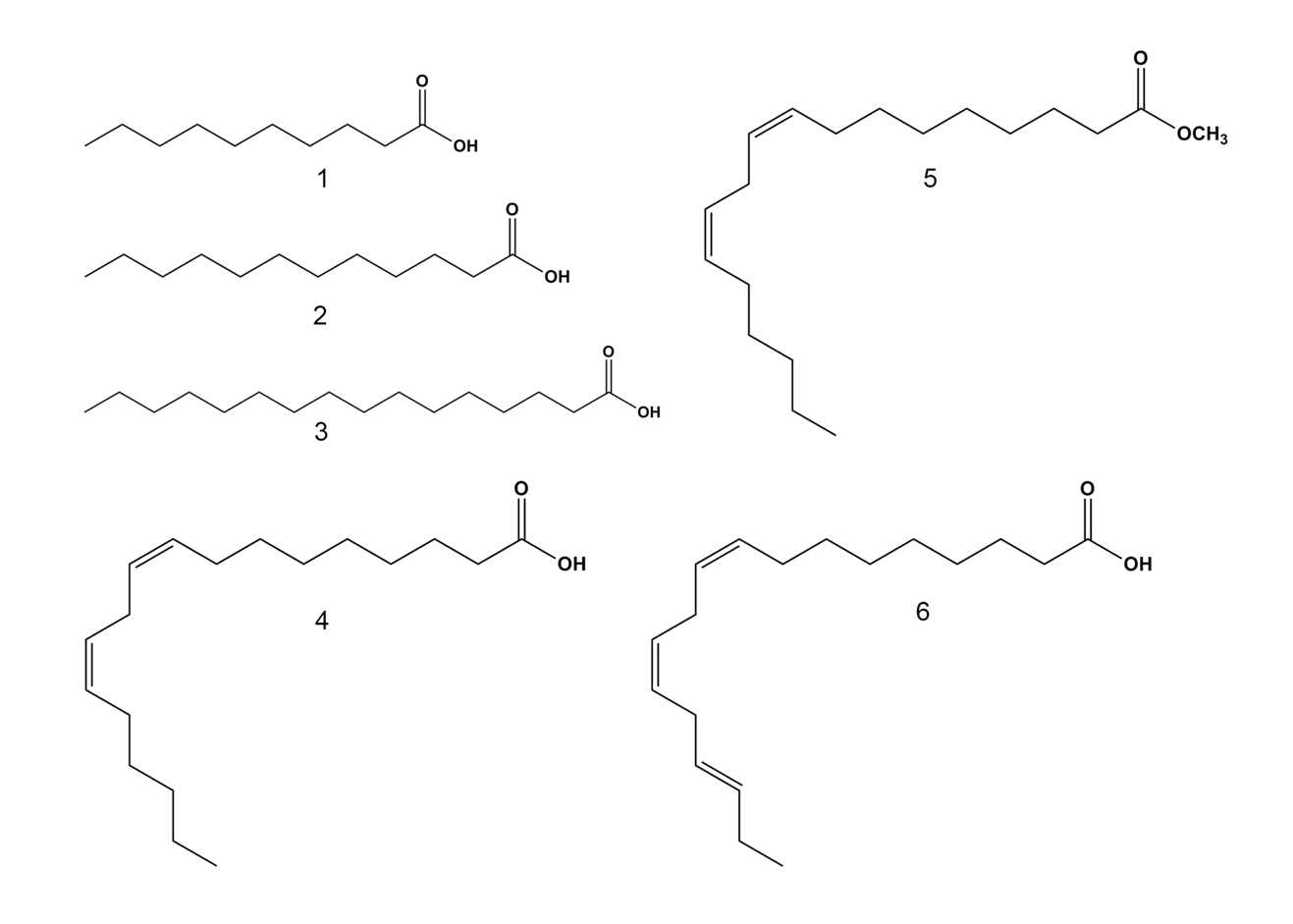


**Supplementary Figure 1.** Structure of HLEA-P1-6 from *H. leucospilota*.


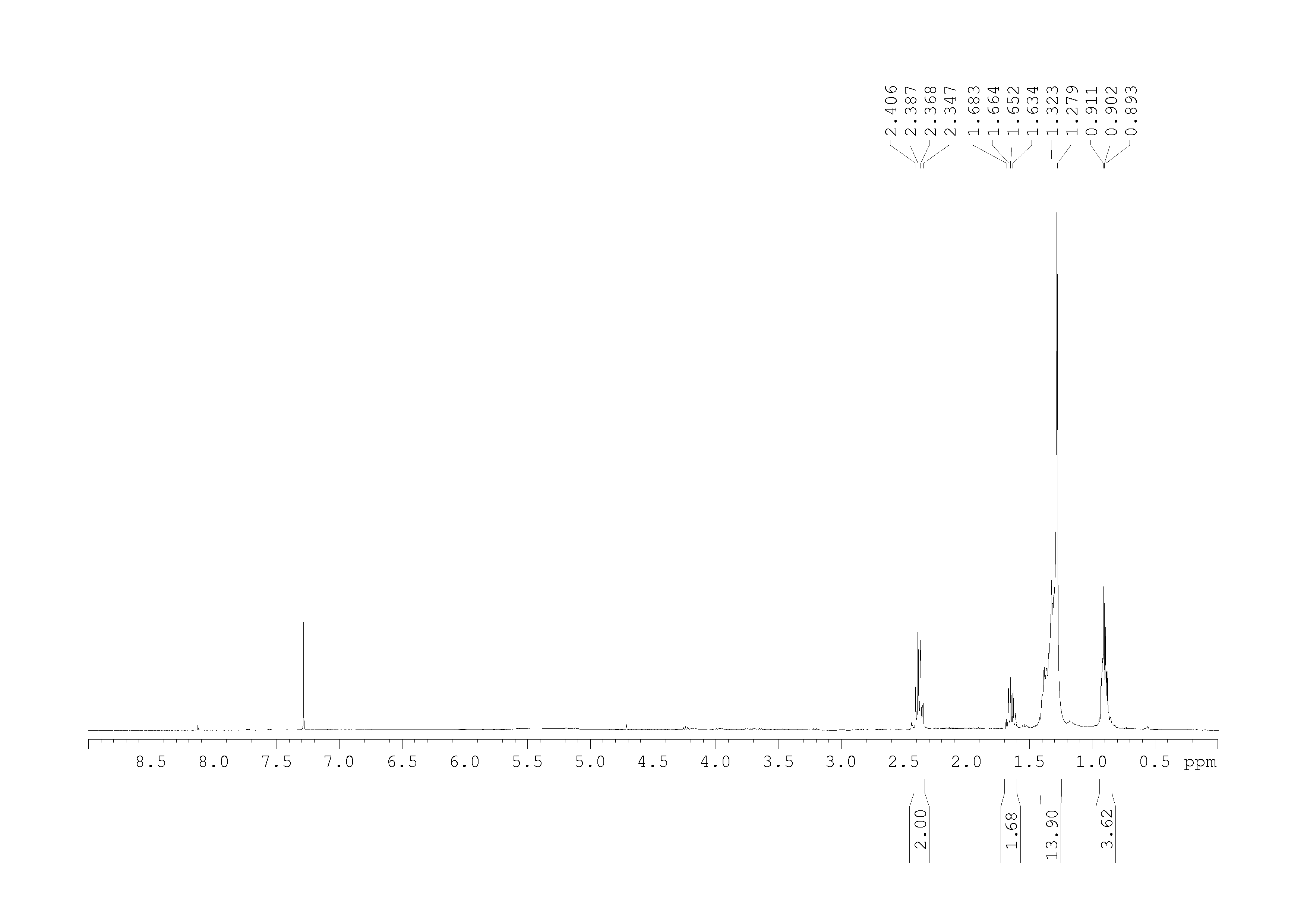


**Supplementary Figure 2.** ^1^H-NMR spectrum of HLEA-P1, Capric acid in CDCl_3_.
